# Supplementary figures and images for: Construction and optimization of multi-platform precision pathways for precision medicine
Source: Sci Rep. 2024 Feb 21;14:4248. doi: 10.1038/s41598-024-54517-8 (PMC10879206; doi:10.1038/s41598-024-54517-8)

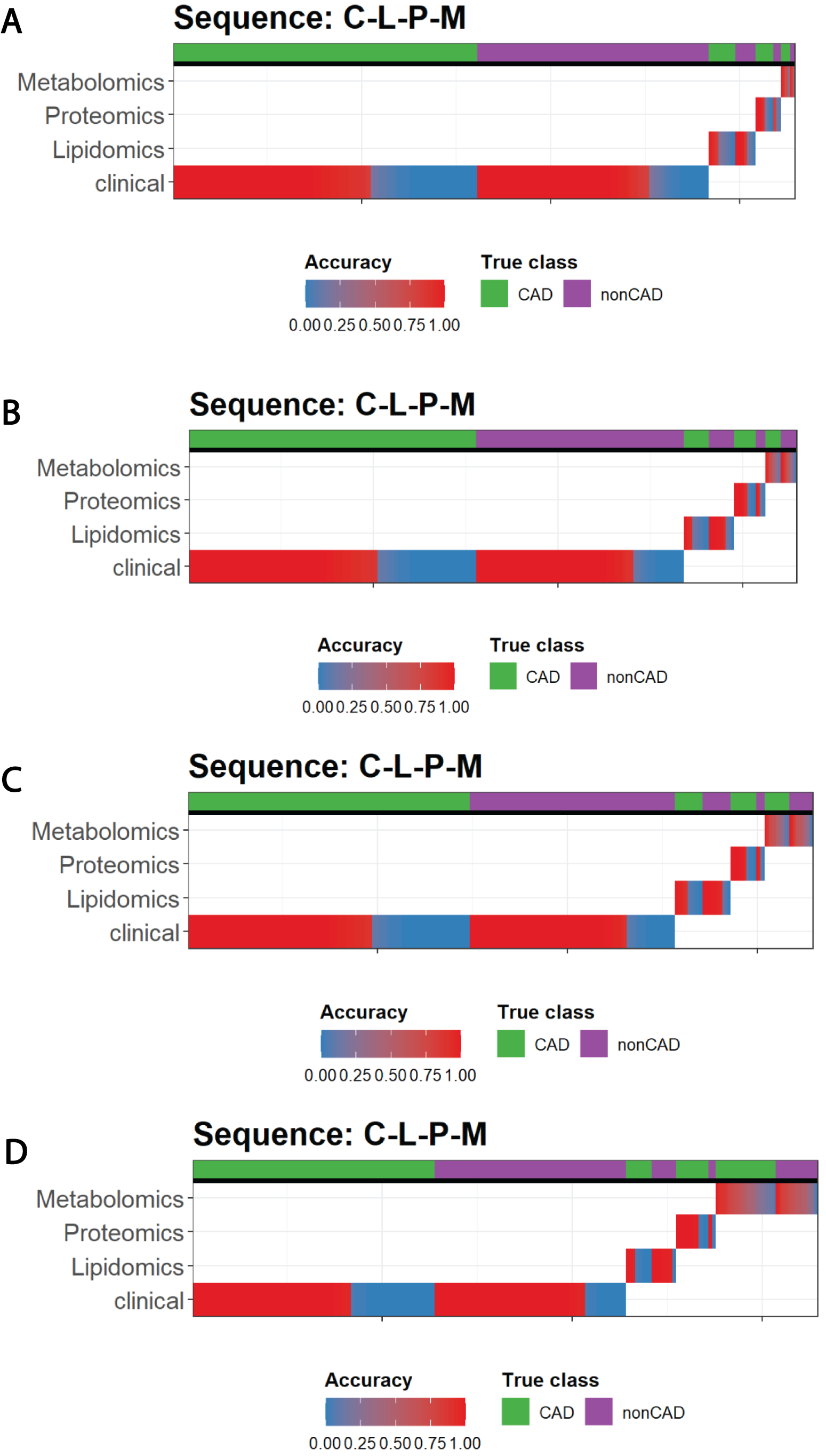

Supplement: Supplementary file 1 — Supplementary Figure S1. [file 41598_2024_54517_MOESM1_ESM.tif]

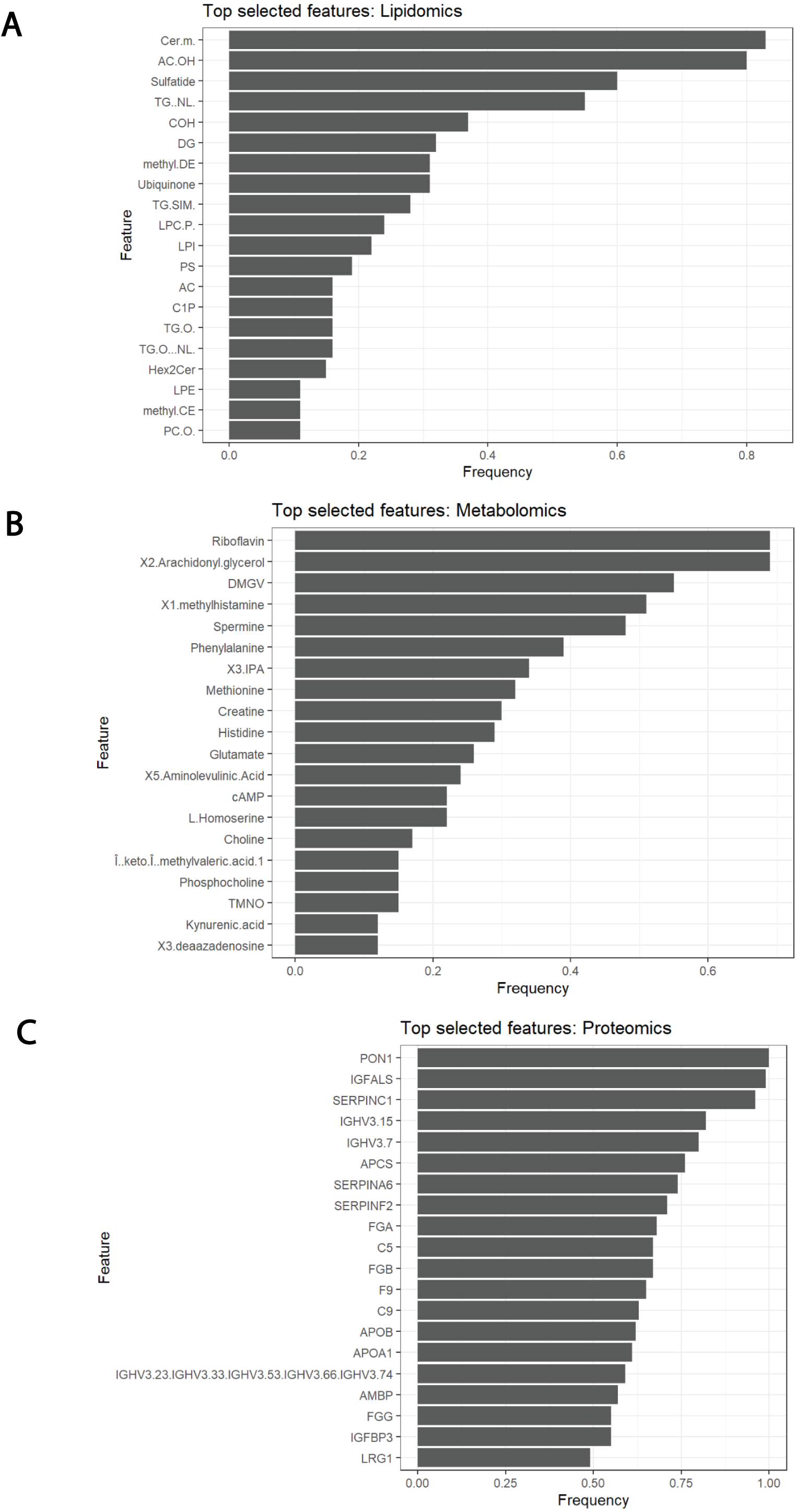

Supplement: Supplementary file 2 — Supplementary Figure S2. [file 41598_2024_54517_MOESM2_ESM.tif]

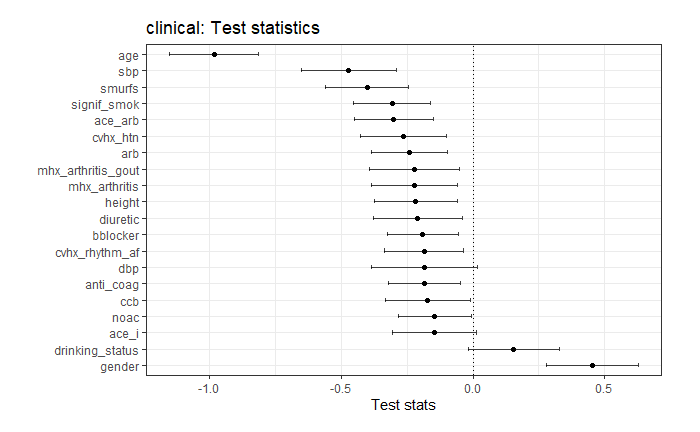

Supplement: Supplementary file 3 — Supplementary Figure S3. [file 41598_2024_54517_MOESM3_ESM.png]

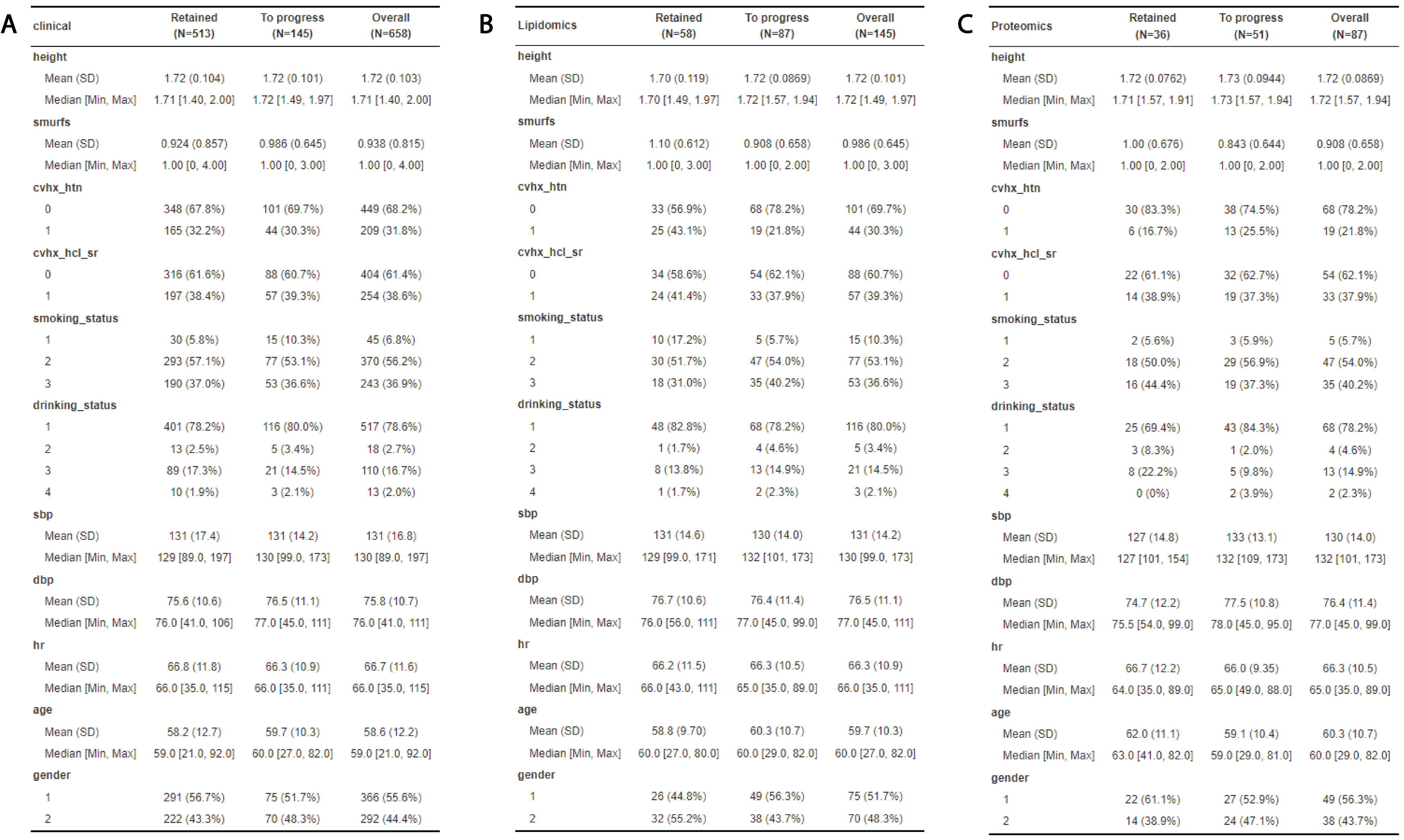

Supplement: Supplementary file 4 — Supplementary Figure S4. [file 41598_2024_54517_MOESM4_ESM.tif]

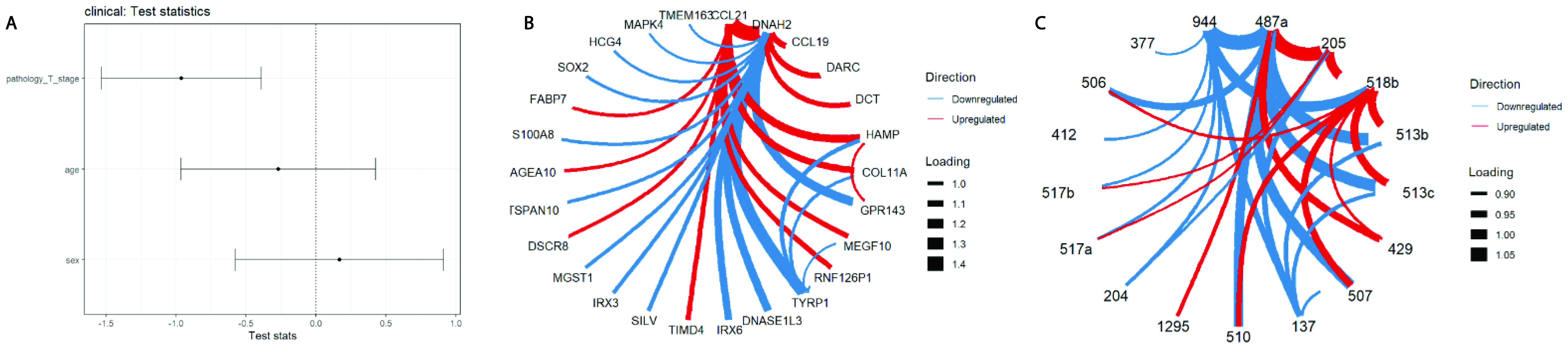

Supplement: Supplementary file 5 — Supplementary Figure S5. [file 41598_2024_54517_MOESM5_ESM.tif]
