# Supplementary material for: Construction and optimization of multi-platform precision pathways for precision medicine
Source: Sci Rep. 2024 Feb 21;14:4248. doi: 10.1038/s41598-024-54517-8 (PMC10879206; doi:10.1038/s41598-024-54517-8)
Supplement: Supplementary file 6 — Supplementary Legends. [file 41598_2024_54517_MOESM6_ESM.docx]

**Supplementary Information**

**Supplementary Table 1:** Clinical characteristics of the BioHEART-CT cohort, categorized by CAD and non-CAD individuals. N represents the total number of individuals in the non-CAD and CAD categories and n represents the number of individuals with the corresponding clinical characteristic. Numerical variables are recorded as Mean (SD) and categorical variables are recorded as n (%).

**Supplementary Table 2:** Balanced accuracy and cost from the sensitivity analysis where the confidence threshold is varied for the clinical-Lipidomics-Proteomics-Metabolomics pathway to classify CAD on the BioHEART-CT cohort.

**Supplementary Figure 1:** Strata plots from the sensitivity analysis where the confidence threshold is varied for the clinical-Lipidomics-Proteomics-Metabolomics pathway to classify CAD on the BioHEART cohort. Strata plots display the accuracy of the patients classified in each stage of the pathway. The x-axis corresponds to each patient, sorted by the platform they were classified in (y-axis), then by their true class (top row), then by accuracy (color). **A**. Strata plot for confidence threshold 0.8. **B**. Strata plot for confidence threshold 0.85. **C**. Strata plot for confidence threshold 0.9. **D**. Strata plot for confidence threshold 0.95.

**Supplementary Figure 2:** Feature selection plots visualize the proportion of folds where each feature was selected, indicative of the importance of each feature to the overall model. **A**. Feature selection plot for Lipidomics in detecting CAD. **B**. Feature selection plot for Metabolomics in detecting CAD. **C**. Feature selection plot for Proteomics in detecting CAD.

**Supplementary Figure 3:** DLDA feature importance plot visualizes the loadings of each feature in the discriminant function, indicative of the importance of each feature. Error bars correspond to a 95% confidence interval for the loadings, estimated from the repeated cross-validation. The above results plot the values for clinical data in detecting CAD. age = age in years, sbp = systolic blood pressure, smurfs = standard modifiable risk factors, signif_smok = smoking pack year history > 10 years, ace_arb = ACE inhibitor/ARB, cvhx_htn = hypertension, arb = ARB, mhx_arthiritis_gout = gout, mhx_arthiritis = osteoarthritis, height = height in meters, diuretic = diuretic, bblocker = beta-blocker, cvhx_rhythm_af = atrial fibrillation, dbp = diastolic blood pressure, anti_coag = anti-coagulant, ccb = calcium channel blocker, noac = NOAC, ace_i = ace inhibitor, drinking_status = drinking status (current, ex-drinker, never), gender = sex (male, female).

**Supplementary Figure 4:** Cohort summary tables summarize the cohorts that are classified or progressed at each stage of the pathway. **A**. Cohort summary table for stage 1 (clinical). **B**. Cohort summary table for stage 2 (lipidomics). **C**. Cohort summary table for stage 3 (metabolomics).

**Supplementary Figure 5:** Feature importance plots for each model built on the TCGA dataset for melanoma prognosis. For models built on log ratios between pairs of features, the DLDA discriminant function feature loadings are visualized as a connection between pairs of features. The thickness of connection is proportional to the magnitude of feature loadings, and color corresponds to the sign of the feature loading. **A**. DLDA Feature importance plot of clinical data. **B**. DLDA Feature importance plot of mRNA data (log ratios). **C**. DLDA Feature importance plot of microRNA data (log ratios).
