# Supplementary material for: Construction and optimization of multi-platform precision pathways for precision medicine
Source: Sci Rep. 2024 Feb 21;14:4248. doi: 10.1038/s41598-024-54517-8 (PMC10879206; doi:10.1038/s41598-024-54517-8)
Supplement: Supplementary file 7 — Supplementary Tables. [file 41598_2024_54517_MOESM7_ESM.docx]

### Supplementary Tables

**Supplementary Table 1:** Clinical characteristics of the BioHEART-CT cohort, categorized by CAD and non-CAD individuals. N represents the total number of individuals in the non-CAD and CAD categories and n represents the number of individuals with the corresponding clinical characteristic. Numerical variables are recorded as Mean (SD) and categorical variables are recorded as n (%).

|  | **Non-CAD**  **N = 279** | **CAD**  **N = 379** |
| --- | --- | --- |
| **Age** | 52.4 (11.3) | 63.1 (10.8) |
| **Sex** |  |  |
| Male | 120 (43.0%) | 246 (64.9%) |
| Female | 159 (57.0%) | 133 (35.1%) |
| **BMI** | 26.3 (4.82) | 27.2 (4.69) |
| **Hypertension** | 69 (24.7%) | 140 (36.9%) |
| **Diabetes** | 12 (4.3%) | 25 (6.6%) |
| **Hypercholesterolemia** | 98 (35.1%) | 156 (41.2%) |
| **Smoking status** |  |  |
| Current smoker | 18 (6.5%) | 27 (7.1%) |
| Never smoked | 170 (60.9%) | 200 (52.8%) |
| Ex-smoker | 91 (32.6%) | 152 (40.1%) |

###

**Supplementary Table 2:** Balanced accuracy and cost from the sensitivity analysis where the confidence threshold is varied for the clinical-Lipidomics-Proteomics-Metabolomics pathway to classify CAD on the BioHEART-CT cohort.

| Confidence Threshold | Balanced Accuracy | Total Cost |
| --- | --- | --- |
| 0.8 | 0.673 | $27665 |
| 0.85 | 0.672 | $31450 |
| 0.9 | 0.682 | $34280 |
| 0.95 | 0.691 | $42620 |

###

### 
